# The Physiological Significance of Endothelial M3 Muscarinic Receptors During Exercise

**DOI:** 10.1161/CIRCRESAHA.125.326589

**Published:** 2025-07-16

**Authors:** Gareth L. Ackland, Patrick S. Hosford, Ana Gutierrez del Arroyo, Alla Korsak, Asif Machhada, Jack Pickard, Genoveva Gomez Gomez De La Torre, Daniel J. Stuckey, Andrew Tinker, Andrew B. Tobin, Jongrye Jeon, Jürgen Wess, Alexander V. Gourine

**Affiliations:** 1 Translational Medicine and Therapeutics, William Harvey Research Institute, Barts and The London School of Medicine and Dentistry, Queen Mary, University of London, London, United Kingdom; 2 Centre for Cardiovascular and Metabolic Neuroscience, Neuroscience, Physiology and Pharmacology, University of London, London, United Kingdom; 3 Centre for Advanced Biomedical Imaging, Division of Medicine, University of London, London, United Kingdom; 4 Clinical Pharmacology and Precision Medicine, William Harvey Research Institute, Barts and The London School of Medicine and Dentistry, Queen Mary, University of London, London, United Kingdom; 5 Advanced Research Centre, 11 Chapel Lane, University of Glasgow, Glasgow G11 6EW, United Kingdom; 6 Molecular Signaling Section, Laboratory of Bioorganic Chemistry, NIDDK, NIH, Bethesda, MD, USA

**Keywords:** Autonomic nervous system, endothelium, exercise capacity, muscarinic receptors, vagus nerve, Autonomic Nervous System, Mechanisms, Physiology, Vascular Biology

## Nonstandard Abbreviations and Acronyms

Exercise capacity is critically dependent on cardiovascular and respiratory responses orchestrated by the autonomic nervous system to support the metabolic demands of physical activity. Higher exercise capacity is strongly associated with a lower resting heart rate, an indirect marker of increased parasympathetic (vagal) tone, while vagal autonomic dysfunction is linked to impaired exercise tolerance.^1^ However, the mechanisms by which vagal parasympathetic activity modulates exercise capacity remain unclear. The parasympathetic nervous system regulates organ/tissue function via its main transmitter acetylcholine acting predominantly via M2 (M2 mAChR) and M3 muscarinic (M3 mAChR) receptors. In this study, we investigated the role of M2 mAChR- and M3 mAChR-mediated signalling in regulating exercise capacity.

Experiments were conducted in accordance with the UK Animals (Scientific Procedures) Act (1986) and institutional approval. Adult male Sprague-Dawley rats and multiple lines of genetically modified mice of both sexes were used. Exercise capacity (expressed as work done in joules) was assessed using a single-lane treadmill following a three-day recruitment/acclimatization protocol, as described.^2^ In anaesthetised (isoflurane) animals, intraperitoneal administration of the β_1_-adrenoceptor agonist dobutamine (0.25-0.5 mg.kg^-1^) was used to mimic the chronotropic and inotropic cardiac responses to increased sympathetic activity during exercise.^2^ Dynamic left ventricular function was quantified using high-resolution echocardiography using Simpson’s rule to approximate left ventricular volume (FUJIFILM Visualsonics Vevo 2100/3100), with simultaneous recording of heart rate (HR).^2^ All assessments were performed blinded to genotype/treatment.

In male rats (2-3 month old), experimentally-induced vagal parasympathetic dysfunction, established following unilateral surgical vagotomy, reduced exercise capacity when assessed 5 days after the surgery (Figure [A]), but had no effect on cardiac responses to β_1_-adrenoceptor stimulation with dobutamine (Figure [B]). Systemic blockade of muscarinic receptors with atropine methylnitrate (2 mg.kg^-1^.h^-1^, intraperitoneally for 4h) also reduced exercise capacity (Figure [A]). However, systemic administration of the M2 mAChR-preferring antagonist AF-DX 116 (2 mg.kg^-1^.h^-1^ for 4h) had no effect (Figure [A]).

We next conducted a series of studies in genetically modified mice (genetic background: C57BL/6; ∼3 months old; ∼50% female). Since M2 mAChR is the predominant muscarinic receptor in the heart, we first selectively deleted cardiac M2 mAChR in M2R^flox/flox^ mice, generated via standard injection of genetically engineered embryonic stem cells into mouse blastocysts (Wess, Jeon, unpublished data). Deletion of cardiac M2 mAChR in M2R^flox/flox^ mice was achieved through intravenous administration of tAAV9-cTnT-iCre-WPRE vector (Vector Biolabs) (Figure [C]), which increased heart rate, as assessed by serial non-invasive ECG recordings (by 30 bpm; IQR:18-38; n=8, p<0.001), compared with no significant change (+8 bpm [IQR:-9 to 24], n=8) in mice that received the control AAV9-cTnT-eGFP-WPRE vector. Knockdown of cardiac M2 mAChR had no effect on exercise capacity in this model (Figure [C]). Contractile and heart rate responses to dobutamine were unaffected 3 weeks after cardiac M2 mAChR knockdown (mean difference in cardiac output: 1.6ml (95% confidence intervals:-7.5 to 4.4);p=0.57;n=8/group). These findings argue against a major role for M2 receptors in the heart in optimising exercise capacity.

We next explored the role of M3 receptors using mice with either global M3 mAChR deficiency (M3-KO) or expressing a phosphorylation-deficient mutant M3 mAChR (M3-KI).^3^ Exercise capacity was reduced in both M3-KO mice (Figure [D]) and M3-KI mice (Figure [E]). Unilateral vagotomy or systemic treatment with the M3 mAChR-preferring antagonist 4-DAMP (1,1-dimethyl-4-diphenylacetoxypiperidinium iodide; 2 mg.kg^-1^, intraperitoneally) reduced exercise capacity in wild-type mice, but had no effect in M3-KO or M3-KI mice (Figures [D,E]). There were no differences in contractile and heart rate responses to dobutamine (mean [95% confidence intervals]) between M3-KO (increase in cardiac output:+2.5ml.min^-1^ [-2.3 to 7.3]; HR:+109bpm [56-162]; n=10), M3-KI mice (increase in cardiac output:+3.7ml.min^-1^ [-2.3 to 9.6]; HR:+89 [38-140]; n=5), and their respective wild-type counterparts (increase in cardiac output:+3.4ml.min^-1^ [-1.6 to 8.5]; HR:+106bpm [66-147]; n=5-10). These data suggest that the reduced exercise capacity observed under conditions of genetic disruption of M3 mAChR signaling cannot be attributed to impaired cardiac function.

Since endothelial M3 mAChR mediates the vasodilatory effects of acetylcholine,^4^ we next hypothesized that impaired M3 signalling in the vascular endothelium might be responsible for the reduced exercise capacity of M3-KO and M3-KI mice. To test this hypothesis, we deleted endothelial M3 mAChR (EC-M3-KO) by crossing M3^flox/flox^ mice with B6.Cg-Tg(Tek-cre)1Ywa/J (Tie2-Cre) mice. The resulting reduction in M3 mAChR gene expression in vascular endothelial cells^4^ was associated with markedly lower exercise capacity (Figure [F]). 4-DAMP (2mg.kg^-1^, intraperitoneally) reduced exercise capacity in control M3R^flox/flox^ mice but had no effect in mice with M3 mAChR deletion in vascular endothelium. Cardiac output (Figure [F]) and heart rate responses to dobutamine (increase in HR:+117bpm [57-175] in M3R^flox/flox^;+109 [56-162] in EC-M3-KO) were unaltered by endothelial M3 mAChR deletion.

Our findings demonstrate, for the first time, an important physiological role of M3 mAChRs expressed in vascular endothelial cells.^5^ We propose that M3 mAChRs in the vascular endothelium are critical for ensuring appropriate tissue and systemic haemodynamic responses to support the circulatory requirements of exercise.

## Supplementary Material

Major Resources Table

## Figures and Tables

**Figure:**
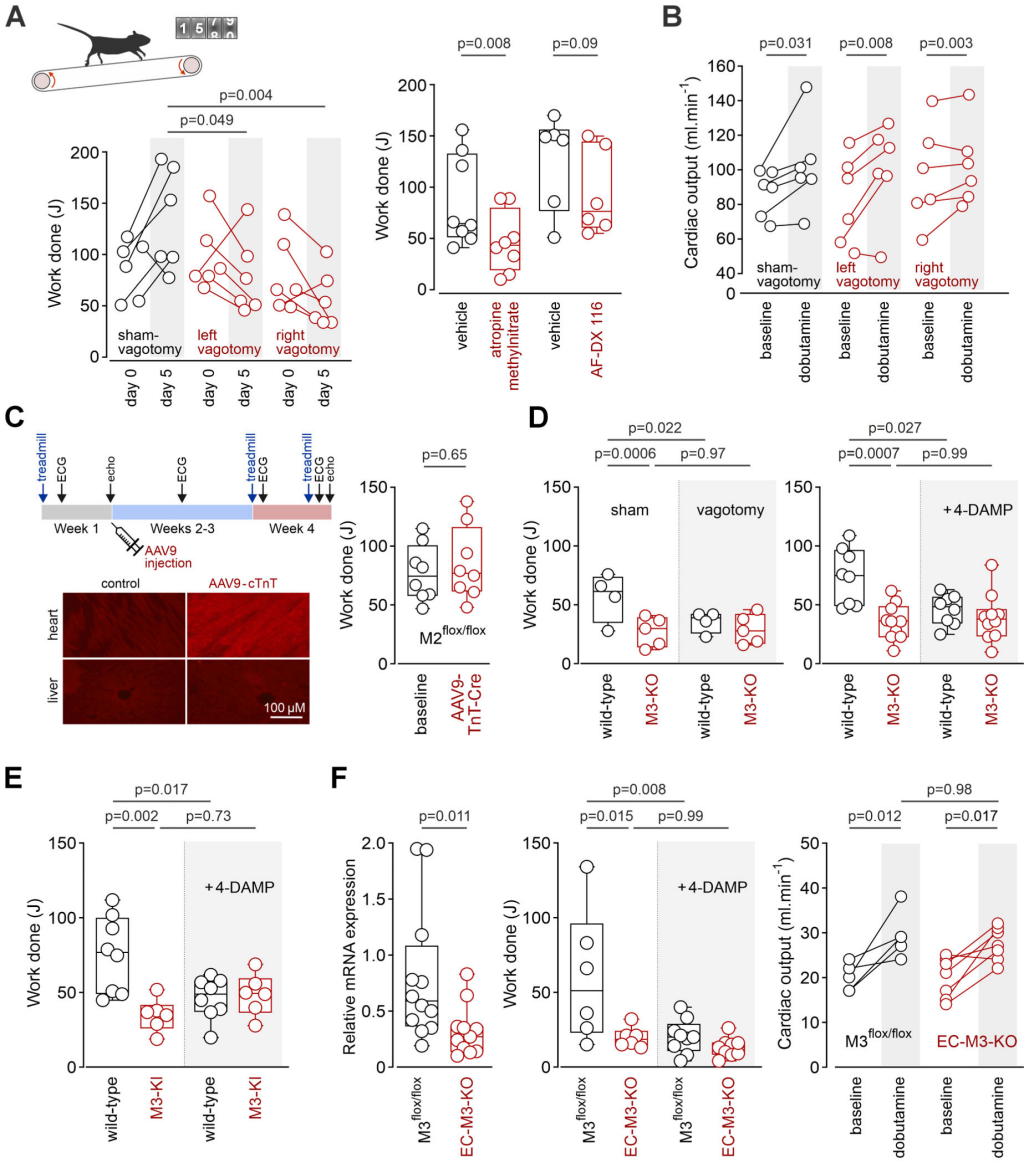
A. In rats exercise capacity decreased 5 days after unilateral vagotomy, in contrast to the increase recorded in sham-operated, treadmill-acclimatized rats. P values: 2-way repeat-measures ANOVA. Systemic treatment with atropine methylnitrate (2 mg kg^-1^ h^-1^) reduced exercise capacity while M2 mAChR-preferring antagonist AF-DX 116 (2 mg kg^-1^ h^-1^) had no effect. *P* values: Wilcoxon signed rank test. B. In rats unilateral vagotomy had no effect on peak increases in cardiac output induced by β_1_-adrenoceptor agonist dobutamine when assessed 5 days after the surgery. *P* values: 2-way repeat measures ANOVA. C. Experimental timeline, illustrating timepoints of exercise testing, ECG recordings, echocardiography and intravenous injections of AAV9-cTnT-GFP-WPRE or AAV9-cTnT-iCre-WPRE in M2^flox/flox^ mice. Images illustrate specific transgene expression in the heart following injections of AAV9-cTnT-GFP-WPRE (viral transduction visualized with anti-GFP immunostaining). Cardiac iCre recombinase expression in M2^flow/flox^ mice had no effect on exercise capacity. *P* value: Mann-Whitney test. D. Reduced exercise capacity in mice with global M3 mAChR deficiency (M3-KO). Unilateral surgical vagotomy or systemic treatment with 4-DAMP (2 mg kg^-1^) reduced exercise capacity in wild-type mice (littermate controls) to the level observed in M3-KO mice, but had no effect in M3-KO mice. *P* values: ANOVA, Sidak’s post-hoc test. E. Reduced exercise capacity in mice expressing a phosphorylation-deficient mutant M3 mAChR (M3-KI). 4-DAMP reduced exercise capacity in wild-type mice but had no effect in M3-KI mice. *P* values: ANOVA, Sidak’s post-hoc test.. F. Reduced vascular M3 mAChR expression and lower exercise capacity in endothelial cell M3 conditional knockout (EC-M3-KO) offsprings of M3^flox/flox^ mice crossed with Tie2-Cre mice. 4-DAMP reduced exercise capacity in M3^flox/flox^ mice, but had no effect in EC-M3-KO mice. Peak increases in cardiac output induced by dobutamine were similar in M3^flox/flox^ and EC-M3-KO mice.*P* values: left graph - Mann-Whitney test; centre and right graphs - ANOVA, Sidak’s post-hoc test.

## Data Availability

Data will be available upon reasonable request.
