# Physiological Significance of Endothelial M3 Muscarinic Receptors During Exercise

**DOI:** 10.1161/CIRCRESAHA.125.326589

**Published:** 2025-07-16

**Authors:** Gareth L. Ackland, Patrick S. Hosford, Ana Gutierrez del Arroyo, Alla Korsak, Asif Machhada, Jack Pickard, Genoveva Gomez Gomez De La Torre, Daniel J. Stuckey, Andrew Tinker, Andrew B. Tobin, Jongrye Jeon, Jürgen Wess, Alexander V. Gourine

**Affiliations:** Translational Medicine and Therapeutics (G.L.A., P.S.H., A.G.d.A., J.P., G.G.G.D.L.T.), William Harvey Research Institute, Barts and The London School of Medicine and Dentistry, Queen Mary, University of London, United Kingdom.; Clinical Pharmacology and Precision Medicine (A.T.), William Harvey Research Institute, Barts and The London School of Medicine and Dentistry, Queen Mary, University of London, United Kingdom.; Centre for Cardiovascular and Metabolic Neuroscience, Neuroscience, Physiology and Pharmacology (P.S.H., A.K., A.M., A.V.G.), University College London, United Kingdom.; Centre for Advanced Biomedical Imaging, Division of Medicine (D.J.S.), University College London, United Kingdom.; Advanced Research Centre, University of Glasgow, United Kingdom (A.B.T.).; Molecular Signaling Section, Laboratory of Bioorganic Chemistry, National Institute of Diabetes and Digestive and Kidney Diseases, National Institutes of Health, Bethesda, MD (J.J., J.W.).

**Keywords:** autonomic nervous system, endothelium, exercise tolerance, receptors, muscarinic, vagus nerve

Exercise capacity is critically dependent on cardiovascular responses orchestrated by the autonomic nervous system to support the metabolic demands of physical activity. Higher exercise capacity is strongly associated with a lower resting heart rate (HR), an indirect marker of increased parasympathetic (vagal) tone, whereas vagal autonomic dysfunction is linked to impaired exercise tolerance.^1^ However, the mechanisms by which vagal parasympathetic activity modulates exercise capacity remain unclear. The parasympathetic nervous system regulates organ/tissue functions via its main transmitter acetylcholine acting predominantly at M2 muscarinic acetylcholine receptors (M2 mAChR) and M3 muscarinic acetylcholine receptors (M3 mAChR). In this study, we investigated the role of M2 mAChR- and M3 mAChR-mediated signaling in regulating exercise capacity.

Experiments were conducted in accordance with the UK Animals (Scientific Procedures) Act (1986) and with institutional approval. Adult male Sprague-Dawley rats and multiple lines of genetically modified mice of both sexes were used. Exercise capacity (expressed as work done in joules) was assessed using a single-lane treadmill after a 3-day recruitment/acclimatization protocol, as described.^2^ In anesthetized (isoflurane) animals, intraperitoneal administration of the β_1_-adrenoceptor agonist dobutamine (0.25–0.5 mg·kg^−1^) was used to mimic the chronotropic and inotropic cardiac responses to increased sympathetic activity during exercise.^2^ Dynamic left ventricular function was quantified using high-resolution echocardiography using the Simpson rule to approximate left ventricular volume (FUJIFILM Visualsonics Vevo 2100/3100), with simultaneous recording of HR.^2^ All assessments were performed by the investigator(s) blinded to genotype/treatment.

In male rats (2–3 months old), experimentally induced vagal parasympathetic dysfunction, established after unilateral surgical vagotomy, reduced exercise capacity when assessed 5 days after the surgery (Figure [A]) but had no effect on cardiac responses to β_1_-adrenoceptor stimulation with dobutamine (Figure [B]). Systemic blockade of muscarinic receptors with atropine methylnitrate (2 mg·kg^−^^1^·h^−^^1^, IP for 4 hours) also reduced exercise capacity (Figure [A]). However, systemic administration of the M2 mAChR-preferring antagonist AF-DX 116 (2 mg·kg^−^^1^·h^−^^1^, IP for 4 hours) had no effect (Figure [A]).

**Figure.**
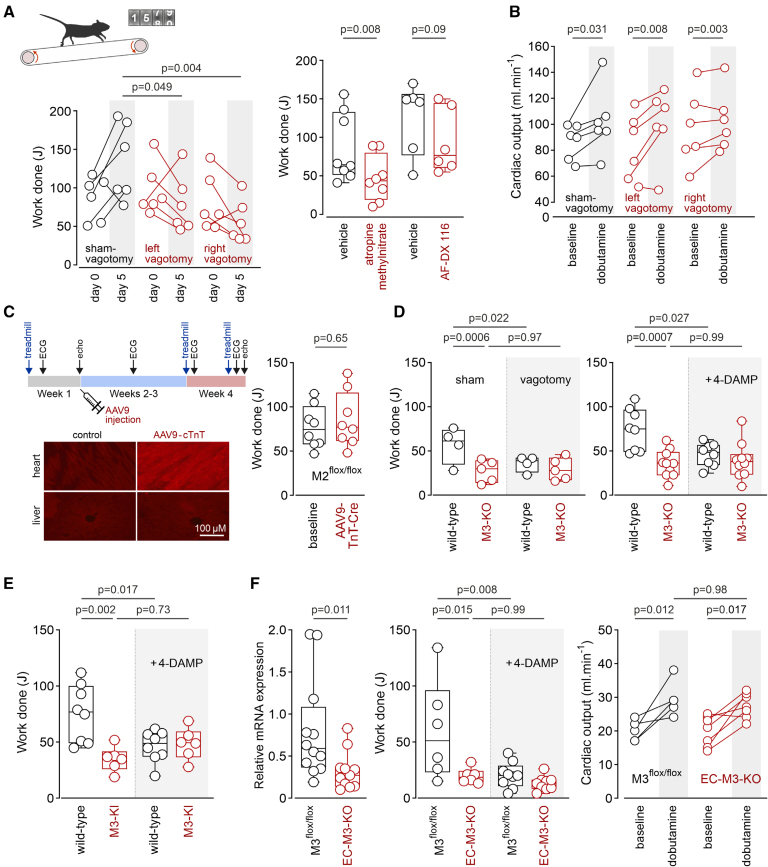
**Endothelial M3 muscarinic receptors determine exercise capacity. A**, In rats, exercise capacity decreased 5 days after unilateral vagotomy, in contrast to the increase recorded in sham-operated, treadmill-acclimatized animals. *P* values: 2-way repeated-measures ANOVA. Systemic treatment with atropine methylnitrate (2 mg kg^−^^1^ h^−^^1^) reduced exercise capacity, whereas M2 mAChR (muscarinic acetylcholine receptor)-preferring antagonist AF-DX 116 (2 mg kg^−^^1^ h^−^^1^) had no effect. *P* values: Wilcoxon signed-rank test. **B**, In rats, unilateral vagotomy had no effect on peak increases in cardiac output induced by β_1_-adrenoceptor agonist dobutamine when assessed 5 days after the surgery. *P* values: 2-way repeated-measures ANOVA. **C**, Experimental timeline, illustrating time points of exercise testing, ECG recordings, echocardiography, and intravenous injections of AAV9 (adeno-associated virus serotype 9)-cTnT (cardiac troponin T)-eGFP (enhanced green fluorescent protein)-WPRE (woodchuck hepatitis virus post-transcriptional regulatory element) or AAV9-cTnT-iCre (improved Cre recombinase)-WPRE in M2^flox/flox^ mice. Images illustrate specific transgene expression in the heart after injections of AAV9-cTnT-eGFP-WPRE (viral transduction visualized with anti-GFP immunostaining). Cardiac iCre recombinase expression in M2^flow/flox^ mice had no effect on exercise capacity. *P* value: Mann-Whitney *U* test. **D**, Reduced exercise capacity in mice with global M3 mAChR deficiency (M3-KO mice [M3 receptor knockout]). Unilateral surgical vagotomy or systemic treatment with 1,1-dimethyl-4-diphenylacetoxypiperidinium iodide (4-DAMP; 2 mg kg^−^^1^) reduced exercise capacity in wild-type mice (littermate controls) but had no effect in M3-KO mice. *P* values: ANOVA, Sidak post hoc test. **E**, Reduced exercise capacity in mice expressing a phosphorylation-deficient mutant M3 mAChR (M3-KI mice [M3 muscarinic receptor knockin]). 4-DAMP reduced exercise capacity in wild-type mice but had no effect in M3-KI mice. *P* values: ANOVA, Sidak post hoc test. **F**, Reduced vascular M3 mAChR expression and lower exercise capacity in endothelial cell M3 conditional knockout (EC-M3-KO mice) offspring of M3^flox/flox^ mice crossed with Tie2-Cre mice. 4-DAMP reduced exercise capacity in M3^flox/flox^ mice but had no effect in EC-M3-KO mice. Peak increases in cardiac output induced by dobutamine were similar in M3^flox/flox^ and EC-M3-KO mice. *P* values: **left** graph: Mann-Whitney *U* test; **center** and **right** graphs: ANOVA, Sidak post hoc test.

We next conducted a series of studies in genetically modified mice (genetic background: C57BL/6; ≈3 months old; ≈50% female). Because M2 mAChR is the predominant muscarinic receptor in the heart, we first used M2R^flox/flox^ mice, generated via standard injection of genetically engineered embryonic stem cells into mouse blastocysts (J. Wess and J. Jeon, 2016, unpublished data). Deletion of cardiac M2 mAChR in M2R^flox/flox^ mice was achieved through intravenous administration of AAV9 (adeno-associated virus serotype 9)-cTnT (cardiac troponin T)-iCre (improved Cre recombinase)-WPRE (woodchuck hepatitis virus post-transcriptional regulatory element) vector (Vector Biolabs; Figure [C]), which increased HR 3 weeks after the injections, as assessed by noninvasive ECG recordings (by 30 beats per minute [bpm]; interquartile range, 18–38; n=8, *P*<0.001), compared with no change (interquartile range, −9 to 24 bpm, n=8) in mice that received the control AAV9-cTnT-eGFP-WPRE vector. Knockdown of cardiac M2 mAChR had no effect on exercise capacity in this model (Figure [C]). Contractile and HR responses to dobutamine were unaffected by cardiac M2 mAChR knockdown (mean difference in cardiac output, 1.6 mL·min^−1^ [95% CI, −7.5 to 4.4]; *P*=0.57; n=8/group). These findings argue against a major role for M2 mAChR in the heart in optimizing exercise capacity.

We next explored the role of M3 receptors using mice with either global M3 mAChR deficiency (M3-KO [M3 receptor knockout]) or expressing a phosphorylation-deficient mutant M3 mAChR (M3-KI [M3 receptor knockin]).^3^ Exercise capacity was reduced in both M3-KO mice (Figure [D]) and M3-KI mice (Figure [E]). Unilateral vagotomy or systemic treatment with the M3 mAChR-preferring antagonist 1,1-dimethyl-4-diphenylacetoxypiperidinium iodide (4-DAMP, 2 mg·kg^−^^1^, IP) reduced exercise capacity in wild-type mice but had no effect in M3-KO or M3-KI mice (Figures [D] and [E]). There were no differences in contractile and HR responses to dobutamine between M3-KO mice (increase in cardiac output, +2.5 mL·min^−^^1^ [95% CI, −2.3 to 7.3]; HR, +109 bpm [95% CI, 56–162]; n=10), M3-KI mice (increase in cardiac output, +3.7 mL·min^−^^1^ [95% CI, −2.3 to 9.6]; HR, +89 bpm [95% CI, 38–140]; n=5), and their respective wild-type counterparts (increase in cardiac output, +3.4 mL·min^−^^1^ [95% CI, −1.6 to 8.5]; HR, +106 bpm [95% CI, 66–147]; n=5–10). These data suggest that the reduced exercise capacity observed under conditions of genetic disruption of M3 mAChR signaling cannot be attributed to impaired cardiac function.

Because endothelial M3 mAChR mediates the vasodilatory effects of acetylcholine,^4^ we next hypothesized that impaired M3 signaling in the vascular endothelium might be responsible for the reduced exercise capacity of M3-KO and M3-KI mice. To test this hypothesis, we deleted endothelial M3 mAChR by crossing M3^flox/flox^ mice with B6.Cg-Tg(Tek-cre)1Ywa/J (Tie2-Cre) mice. The resulting reduction in M3 mAChR expression in vascular endothelial cells was associated with markedly lower exercise capacity (Figure [F]). 4-DAMP (2 mg·kg^−^^1^, IP) reduced exercise capacity in control M3R^flox/flox^ mice but had no effect in mice with M3 mAChR deletion in vascular endothelium. Cardiac output (Figure [F]) and HR responses to dobutamine (increase in HR, +117 bpm [95% CI, 57–175] in M3R^flox/flox^; +109 bpm [95% CI, 56–162] in conditional knockout mice) were unaffected by endothelial M3 mAChR deletion.

Our findings demonstrate, for the first time, an important physiological role for M3 mAChRs expressed in vascular endothelial cells.^5^ We propose that endothelial M3 mAChRs are critical for ensuring appropriate tissue and systemic hemodynamic responses to support the circulatory requirements of exercise.

## ARTICLE INFORMATION

### Author Contributions

G.L. Ackland, P.S. Hosford, A. Gutierrez del Arroyo, A. Korsak, A. Machhada, J. Pickard, G.G. Gomez De La Torre, and D.J. Stuckey conducted the experiments and analyses. J. Jeon, J. Wess, A. Tinker, and A.B. Tobin supplied experimental materials and animals. G.L. Ackland and A.V. Gourine wrote the article with help from the other coauthors.

### Data Availability

Data will be available upon reasonable request.

### Sources of Funding

This study was supported by British Heart Foundation Program grant funding (RG/19/5/34463).

### Disclosures

The authors report no conflicts.

### Supplemental Material

Major Resources Table
