# Supplementary material for: The Physiological Significance of Endothelial M3 Muscarinic Receptors During Exercise
Source: Circ Res. Author manuscript; Available in PMC 2025 Jul 28. (PMC7617941; doi:10.1161/CIRCRESAHA.125.326589)
Supplement: Supplementary file 1 [file EMS206882-supplement-Major_Resources_Table.pdf]

Major Resources Table

Animals (in vivo studies)

Species	Vendor or Source	Background Strain	Sex	Persistent ID / URL
Rat	Charles River	Sprague Dawley	M	https://www.criver.com/products-services/find-model/cd-sd-igs-rat

Genetically Modified Animals

	Species	Vendor or Source	Background Strain	Other Information	Persistent ID / URL
M3R KI	MOUSE	NIH, Tobin lab	C57BL/6	M&F	https://pmc.ncbi.nlm.nih.gov/articles/PMC2889095/#S3
B6.Cg-Tg(Tek-cre)1Ywa/J	MOUSE	JAx	B6	TIE2-CRE; M&F	https://www.jax.org/strain/008863
M2R$^{flox/flox}$	MOUSE	NIH, Wess	C57BL/6	M2Rflox; M&F	Available from Jurgen Wess, NIH
Chrm3^{tm2Jwe}	MOUSE	NIH, Wess	129S6/SvEvTac	M3R FLOX; M&F	https://www.informatics.jax.org/allele/MGI:3770175
B6N.129S6(B6J)-*Chrm3*^{tm1Jwe}/J	MOUSE	NIH, Wess	C57BL/6N	M3R-KO; M&F	https://www.jax.org/strain/030163

Viral vectors

Clone Name	Sequence	Source / Repository Persistent ID / URL
AAV9-cTnT-iCre-WPRE	Vectorbiolabs Cat. No: VB5412 https://www.vectorbiolabs.com/product/vb5412-aav-with-ctnt-promoter-driven-icre/	
AAV9-cTnT-GFP-WPRE	Vectorbiolabs Cat. No: VB5428 https://www.vectorbiolabs.com/product/vb5428-aav-with-ctnt-promoter-driven-egfp/	

Drugs

Name	Vendor or Source	Persistent ID / URL
4-DAMP	Tocris	Cat. No. 0482
AF-DX116	Tocris	Cat. No. 1105/10
Atropine methylnitrate	Cayman Chemical	product/9002272/

PCR primers

Relative expression of M3R in aortic endothelium in each mouse was compared with brain M3 mRNA expression, and using the housekeeping gene cyclophilin.

Name	Vendor or Source	Persistent ID / URL
Cyclophilin	Forward: 5'-GAGCTGTTTGCAGACAAAGTTC-3'<br>Reverse: 5'-CCCTGGCACATGAATCCTGG-3'	http://pga.mgh.harvard.edu/primerbank/<br>PrimerBank ID 6679438c1
M3 mAChR	Forward: 5'-ACCTGTTCACGACCTACATCA-3'<br>Reverse: 5'-AGTGAGTGGCCTGGTAATAGAAA-3'	http://pga.mgh.harvard.edu/primerbank/<br>PrimerBank ID 162287049c2

DOI [to be added]

M3 sense and antisense

ARRIVE guidelines
The experiments were performed in accordance with the UK Animals (Scientific Procedures) Act (1986), with project approval from the UCL and QMUL Animal Care and Use Committees.

Study Design

Groups	Sex	Age	Number (prior to experiment)	Number (after termination)	Littermates/controls (Yes/No)
Sprague-Dawley	M	2-3 mo.	63	0	yes
M2Rflox	M/F	3 mo.	16	0	No- served as own control after AAV9 injection
M3R-KO & WT	M/F	3 mo.	27	0	yes
M3-KI & WT	M/F	3 mo.	13	0	yes
M3flox	M/F	3 mo.	31	0	No
EC-M3-KO	M/F	3 mo.	33	0	M3flox

Sample Size: Based on our previous work in rats where genetic silencing of the largest population of brainstem vagal preganglionic neurons residing in the dorsal vagal motor nucleus reduced exercise capacity by >70%,[1] we calculated that at least 6 mice per group were required to have a 90% chance of detecting, as significant at the 5% level, a decrease in the primary outcome measure from 100±25J in the control group to 50±25J in the experimental group.

Randomization
The order of experiments was determined using https://randomizer.org. No animals were excluded from the study. Because it is well established that different mouse strains and lines exhibit variable exercise capacities,[2,3] no mice were excluded on the basis of low exercise capacity during the period of treadmill acclimatization.

Blinding
All analysis was conducted blinded to genotype by at least one blinded analyzer (JP, GDLT, AGDA, DS). The identify of AAV9 vectors were masked by being labelled as vials A or B, for administration and final analysis.

References

1. Machhada A, Trapp S, Marina N, Stephens RCM, Whittle J, Lythgoe MF, Kasparov S, Ackland GL, Gourine AV. Vagal determinants of exercise capacity. *Nat Commun*. 2017;8:15097.
2. Massett MP, Berk BC. Strain-dependent differences in responses to exercise training in inbred and hybrid mice. *Am J Physiol Regul Integr Comp Physiol*. 2005;288:R1006-1013.
3. Avila JJ, Kim SK, Massett MP. Differences in Exercise Capacity and Responses to Training in 24 Inbred Mouse Strains. *Front Physiol*. 2017;8:974.

DOI [to be added]
